# How does community context influence coalitions in the formation stage? a multiple case study based on the Community Coalition Action Theory

**DOI:** 10.1186/1471-2458-10-90

**Published:** 2010-02-23

**Authors:** Michelle C Kegler, Jessica Rigler, Sally Honeycutt

**Affiliations:** 1 Emory Prevention Research Center, Rollins School of Public Health, Emory University, 1518 Clifton Road, NE, Atlanta, GA 30322, USA; 2 Arizona Department of Health Services, 150 N. 18th Ave, Suite 120, Phoenix, AZ 85007, USA

## Abstract

**Background:**

Community coalitions are rooted in complex and dynamic community systems. Despite recognition that environmental factors affect coalition behavior, few studies have examined how community context impacts coalition formation. Using the Community Coalition Action theory as an organizing framework, the current study employs multiple case study methodology to examine how five domains of community context affect coalitions in the formation stage of coalition development. Domains are history of collaboration, geography, community demographics and economic conditions, community politics and history, and community norms and values.

**Methods:**

Data were from 8 sites that participated in an evaluation of a healthy cities and communities initiative in California. Twenty-three focus groups were conducted with coalition members, and 76 semi-structured interviews were conducted with local coordinators and coalition leaders. Cross-site analyses were conducted to identify the ways contextual domains influenced selection of the lead agency, coalition membership, staffing and leadership, and coalition processes and structures.

**Results:**

History of collaboration influenced all four coalition factors examined, from lead agency selection to coalition structure. Geography influenced coalition formation largely through membership and staffing, whereas the demographic and economic makeup of the community had an impact on coalition membership, staffing, and infrastructure for coalition processes. The influence of community politics, history, norms and values was most noticeable on coalition membership.

**Conclusions:**

Findings contribute to an ecologic and theory-based understanding of the range of ways community context influences coalitions in their formative stage.

## Background

Thousands of communities have formed coalitions over the past 20 years to tackle a wide range of public health and social issues through collaboration [1-4]. As coalitions have become commonplace in health promotion practice, the literature has grown considerably, as have the number of theories and conceptual models of coalition behavior [5-9]. One of these theories, the Community Coalition Action Theory (CCAT), includes a series of "practice-proven" propositions that summarize what is known empirically and what is commonly believed about how community coalitions can lead to improved health and social outcomes [10]. One of its propositions is that community context, including history of collaboration, geography, demographics, and local norms and values, influence coalition functioning and outcomes at each stage of coalition development.

This proposition is strongly supported in the wisdom literature, and to a lesser extent empirically [11-13]. Given that coalitions are rooted in complex and dynamic community systems, it is intuitive that external forces within the community at least partly shape coalitions. Despite several calls for case study research on the topic, relatively few studies have attempted to systematically document how context impacts coalitions [14,15]. Although many researchers recognize the need for theory-based research on coalitions, including contextual influences on coalitions, much of the research conducted to date lacks an underlying theoretical framework [6,16]. The current study is one of the first theory-based studies to systematically examine how community context influences coalitions in the formation stage of development.

Coalition theories and models, including the CCAT, suggest that coalitions develop in stages [10,17,18]. Movement through the stages is not always linear because coalitions can cycle back to earlier stages as they take on new issues, recruit new members or update action plans. The CCAT identifies three stages: formation, maintenance and institutionalization. Formation is the first stage, and associated tasks focus on creation of a new collaborative entity or reconstitution of an existing collaborative structure into a more formal coalition. Basic tasks in the formation stage include convening a core group of coalition members, typically with a strong and shared interest in the mission of the coalition [10,17]. This core group then mobilizes and recruits coalition members who represent a broad cross-section of the community, including professionals who work in the community, residents who represent themselves or various constituencies such as parents or neighborhoods, and individuals who both live and work in the community [19]. Identification of staff and coalition leaders also takes place in the formation stage. Staff are usually employed by the lead agency or group that convenes the coalition and may or may not also serve in a leadership capacity for the coalition. Important leadership functions that take place in the formation stage include the establishment of an organizational structure and processes that guide coalition functioning in communication, decision-making, and conflict resolution. In a study of seven asthma coalitions, Butterfoss et al. documented that the formation stage took an average of 12 months and was heavily influenced by the level of experience partner organizations had in working together in a coalition prior to the new initiative [20].

Commonly mentioned contextual factors with the potential to influence coalitions in the formation stage include geography, history of collaboration, economics, political climate, and community readiness [7,10,21]. These factors create the backdrop in which a coalition operates and intuitively have the potential to impact coalitions in a variety of ways. The handful of studies examining context generally describe the history of collaboration or community readiness prior to coalition formation. Butterfoss et al., for example, describe how asthma coalitions were formed out of existing collaborative relationships in order to respond to a specific funding opportunity [20]. Similarly, Nezlek and Galano found that most of the teen pregnancy prevention coalitions they observed were also formed out of pre-existing collaborative relationships [22].

Community readiness assessments have been done in a range of communities on a broad array of topics, often as a precursor to coalition development [23-26]. These projects used the Community Readiness Model developed by Edwards et al., which views community readiness as topic-specific, with nine stages and six dimensions such as leadership support for prevention efforts and community climate or attitudes toward the issue [27]. Feinberg et al. explicitly examined correlations between community readiness, coalition functioning and perceived effectiveness in 21 coalitions targeting adolescent problem behavior [28]. At the coalition level, community readiness was correlated with both internal functioning and perceived effectiveness. In discussing their findings, the authors highlighted infighting as a possible explanation for how community readiness, or lack thereof, may impede a community's ability to establish an effective coalition.

Of the relatively few case studies that examined community context, each studied different dimensions of context in relation to different outcomes, so it is difficult to synthesize findings across studies. In a multiple case study of three substance abuse prevention coalitions, Reininger et al. documented how mistrust between groups negatively affected coalition formation, particularly through challenges in leadership and staffing [29]. In a multiple case study of coalition factors affecting implementation, Kegler et al. observed how community politics around tobacco control in a tobacco-producing state influenced coalition formation and implementation by limiting who joined the coalition and restricting the range of possible action strategies [30]. Based on their observations as evaluators, Wandersman and colleagues documented how historical racial tensions, geographic and political divisions, and competing prevention initiatives impacted a range of coalition variables, including structure, process, leadership and planning [15].

The purpose of this paper is to examine how community context influenced coalition formation in eight communities participating in a broad-based healthy communities initiative that required the formation of a collaborative governance structure. This is one of the only studies to purposely focus on community context and its impact on explicit coalition factors as identified in a specific theory. This study examines how history of collaboration, community politics and history, community norms and values, community demographics and economic conditions, and geography influence key CCAT constructs associated with coalition formation: lead agency selection, staffing and leadership, coalition membership, coalition process and coalition structure.

## Methods

### Description of the CHCC Program and Evaluation

This study is a secondary analysis of data from an evaluation of the California Healthy Cities and Communities (CHCC) Program. The original evaluation was designed to document the process of community development in 20 participating communities and assess changes that resulted from the local initiatives [31,32]. Participating communities were selected through a competitive process and were awarded a total of $125,000 over a three-year period; three distinct cohorts participated, beginning in 1998 and ending in 2003. During the initial planning year, each community developed a broad-based and multi-sectoral governance structure or coalition, produced a shared vision, conducted an asset-based community assessment, identified a priority community improvement focus, and developed an action plan with goals, objectives and evaluation plans. The subsequent two years were spent implementing the community action plans. Nine of the 20 sites were designated as primary evaluation sites and participated in more extensive data collection activities, including two rounds of site visits and focus groups. The original evaluation was based on an evaluation framework developed using a participatory approach with local California Healthy Cities coordinators [31]. The current study was conducted as a secondary analysis and did not actively engage evaluation participants. The research protocol (#00002847) was reviewed by the Emory University Institutional Review Board and classified as exempt due to minimal risk to study participants.

### Case Selection

The current study draws upon data from eight of the primary sites because more data were available from the primary sites, including three focus groups per site. These eight sites represent a range of geography, population density, lead agency and demographic characteristics (Table 1). They represent the four types of communities as classified in the larger evaluation study; two are rural regions with multiple small towns, two are municipalities within rural counties, two are municipalities within major metropolitan areas, and two are neighborhoods within major cities. The ninth primary site was another rural region and was excluded from the current analyses to allow for even distribution of cases across community types.

**Table 1:** Description of Case Study Communities

Community	Total Population to nearest 100	Population Density (persons per square mile)	Community Descriptor	Coalition Size in Planning Year
**Rural Communities**				
A	1,300	3.6	Rural region in Northern California	13
B	1,600	5.7	Rural region in Northern California	7
C	5,700	4,042	Rural municipality in the Central Coast region	15
D	19,400	4,522	Rural municipality in the Central Valley	15
**Urban Communities**				
E	50,200	6,440	Urban neighborhood in Southern California	10
F	55,00	2,370	Urban municipality in Southern California	33
G	68,000	12,141	Urban neighborhood in Southern California	42
H	130,000	3,983	Urban municipality in Northern California	16

### Data Collection Methods

The current analysis draws most heavily from focus groups conducted with coalition members and semi-structured interviews with local coordinators and leaders. In all but one site, three focus groups were conducted with active coalition members over the course of the evaluation, two in the planning year and one at the end of the project period (23 focus groups, n = 162). The focus groups discussed major accomplishments, the community development process, and factors that facilitated and inhibited their progress. They also described their communities in terms of important or active organizations and groups, community sectors, divisions within the community, and community strengths upon which they were able to build.

A total of 76 in-depth interviews were conducted with representatives from the eight sites reported on here. Detailed interview guides were developed for each year of the three-year grant period with interviews conducted via telephone or in-person depending on the year. Interviews were conducted with the local coordinators of each coalition at four points in time. Coalition leaders, selected by the coordinators as active in and knowledgeable about the coalitions, and lead agency directors were also interviewed, both in the planning year and at the end of the project period. Coalition coordinators and lead agency directors were generally the same individuals over time; coalition leaders often differed between the planning and implementation phase interviews. A series of questions from the planning phase interview guide examined the community development process, including initial community mobilization, resident involvement and governance structures. The implementation phase interview guides focused on community capacity and community change outcomes. Analysis for the current study included all interviews from all three years; however, the majority of the findings relevant to coalition formation emerged from the first year interviews.

### Data Analysis

The interviews and focus groups were tape-recorded and transcribed verbatim. For the current study, case study data were analyzed one case at a time; data consisted of all transcripts from the focus groups and interviews pertaining to that case. The theoretical constructs from the Community Coalition Action Theory were used as an organizing framework [10]. Contextual domains, originally derived from the literature and CCAT, were further defined after analysis of the first case. Table 2 lists the contextual domains included in this analysis, along with a brief explanation and an illustrative quote for each domain. For each contextual domain, analysts independently reviewed all of the transcripts to identify passages of text that suggested a contextual influence on the coalition. Analysts then placed these passages in a series of matrices organized by contextual variable (e.g., geography) and CCAT construct (e.g., coalition membership), thus creating an audit trail to increase the reliability of case study findings [33,34]. The resulting series of matrices (one for each contextual feature) constituted the case records for each site. Each analyst then identified tentative themes related to each contextual factor through a series of implicit questions about relationships between the contextual factor and relevant CCAT constructs. For example, how does geography influence selection of a lead agency for coalition-based efforts, if at all? Upon identification of tentative themes for each site, the study team met to review the case study and possible themes specific to that case. This process was completed for all eight cases. For the first two cases, all three members of the study team completed the in-depth process in order to achieve a common understanding of the analytic approach. For the subsequent six cases, two analysts completed the in-depth process.

**Table 2:** Description of Contextual Domains

Domains	Explanation of domain	Example quote
History of collaboration	Prior experience of coalition members in working together and with others in the community. Includes both organizational collaboration and interpersonal networks and relationships.	"It's usually the same agencies, and that primarily is because, well one, they're the ones willing to play together, and two, they're the agencies in the community that have administrations that's willing, able to sit at a table and put together a proposal or something." *- Local coordinator*
Community politics and history	Formal and informal divisions, factions and groups within the community, and how these groups interact--or do not interact--with one another. History refers to community-wide shared events that occurred before the coalition began, particularly large events that shaped or influenced the community.	This used to be a stage road, and the stages used to get robbed, and ever since then, we've had a bad reputation. [...] Everybody around here who, needed to look down on someone, whoever those folks were, this was the place they talked about and it probably got a worse reputation than it deserved. *-Focus group of coalition members*
Community norms and values	Social norms, beliefs, and values prevalent in the community.	"One of the norms is sort of the ferocious independence. It's not very easily breached....If I was going to kind of put one overarching goal on everything that we've done funded by whoever, it's that we're trying to create a community that has the attitude and awareness that we're all responsible for all of our children, and we're going to do as good a job as we can, so those norms and community values have been very challenging, and are being slow to change but are and will." *-Local coordinator*
Community demographics and economic conditions	The educational, racial/ethnic, and socio-economic makeup of the community. Also includes factors related to the local economy, such as employment opportunities and business presence within the community.	"I think that the economy seems to have a lot to do with the problems that we have. The people that we're working with are, to a large extent, they're economically depressed." *- Local coordinator*
Geography	Conditions that result from the community's geography, particularly differences observed between urban and rural communities. Also includes issues related to how the CHCC community is situated within a larger county or metropolitan area.	"I think one of the weaknesses here of living in this particular town is, that we, geographically speaking, we're like a forgotten town, because anything that comes our way never gets here. Because we're at the end of --- County and --- County, everything just travels in that direction and the other direction, not in between. And I have seen that, I have seen that whenever we get something started, it's really a struggle to keep it, and to get it. They don't really know we're here." *- Coalition leader*

After completing the analysis of individual cases, the study team conducted a cross-case analysis by first consolidating all the findings related to each individual contextual factor [35]. For example, a single document was created to contain all the site-specific findings related to geography. Each analyst identified tentative statements, organized by CCAT construct, that accounted for the patterns across cases (observed in ≥ 2 sites) and documented supporting evidence. The analysts then met to compare statements. When statements were similar for both analysts, these were designated as a theme. When analysts identified different themes, a discussion ensued and consensus was used to designate a statement as a theme or not. The resulting cross-site patterns and themes are reported here, with examples from specific cases to illustrate selected findings.

## Results

### Lead Agency Selection

The lead agency is the group that convenes the coalition in response to an opportunity, threat or mandate. Table 3 lists major contextual themes related to selection of the lead agency. Of the five contextual factors examined, history of collaboration was the most influential on the emergence of the lead agency. A strong theme was that organizations that were the lead agency in a prior related project were positioned to emerge as the lead agency for a new collaborative project. Several sites, for example, already had funding for Healthy Start initiatives. The CHCC funding appeared compatible with the missions of the lead agencies involved in Healthy Start, so they applied for CHCC funding and remained as lead agencies for the new initiative. Several other sites had recently completed community visioning or assessment projects and the lead agencies for these community-building efforts took the lead role for the CHCC project as well.

**Table 3:** Themes Related to Contextual Influences on Lead Agency, Leadership and Staffing Selection

Coalition Factor and Related Themes	History of Collaboration	Community Politics and History	Community Norms and Values	Community Demographics and Economic Conditions	Geography
**Lead Agency**					
Organizations that served as the lead agency in similar projects in the past tend to remain as lead agencies for new projects.	**X**				
**Leadership and Staffing**					
Individuals who have served as staff and/or leaders in similar past projects tend to remain in those positions in new initiatives.	**X**				
Community diversity can affect who is selected as staff when needed skill sets include cultural competence and language abilities.				**X**	
Rural areas with small populations may have a limited pool of people with the skills needed to effectively staff a collaborative initiative.					**X**
The lack of agency professionals in rural areas can necessitate greater volunteer involvement in coalition staff and leadership roles.					**X**

### Leadership and Staffing

Leadership and staffing refers to interpersonal and organizational skills shared by leaders and staff to facilitate coalition functioning. Leadership and staffing, more specifically who was selected as coalition staff and/or leader, was influenced by history of collaboration, community demographics and economic conditions, and geography (Table 3). History of collaboration affected who would be selected as staff in that individuals who had served in a staff role in past similar projects tended to remain as staff for the new initiative. Community demographics and economic characteristics also affected who was hired as program staff because staff members often needed to speak the language and/or understand the culture of the diverse groups within a geographic community. For example, one site hired Latina lay health advisors, Promotoras, to complete community assessment activities and another site hired tutors with diverse ethnic backgrounds to match those of the youth who were being served.

Geography influenced leadership and staffing most noticeably in the rural areas. Several sites noted that in rural areas with a small population, the pool of talented staff was small. A coordinator from a rural region commented that if "*you'd put an ad in the paper, you won't even get anybody to apply*." Another coordinator explained, "*I feel like we have remained completely committed to hiring locally which is totally in line with our mission .... It's also been a challenge, in that sometimes the people that were available locally to hire didn't have the set of skills already that was needed in the position ...it's been an on the job training kind of a process*." Others commented that in rural areas, people with useful skills often commuted to nearby larger towns for better jobs and better pay.

Also related to geography, key staff or leaders who decreased their involvement for personal reasons often had a big impact on small town coalitions due to the small number of additional residents who could step in to fill the gap. One coordinator commented, "*in a small community like this, a few people having something go on can really impact what's happening, like for instance [name] losing her mom and having to go away for a couple months*." The study team also noted that residents had to volunteer more in rural areas because there were so few agency personnel who could take on staffing or leading a coalition as part of their professional responsibilities. A coordinator explained, "*the time has been really inadequate in terms of paid time ... because there's nobody else, and I know how it functions in cities, I'm guessing there's more people to take on a piece of it as part of their job, their paid employment, and here that has not been the case*."

### Coalition Membership

Coalition membership was strongly influenced by contextual factors, with themes emerging for each of the five contextual domains examined. Table 4 presents major themes related to coalition membership by domain. Two themes were related to the history of collaboration between people and organizations in the community. First, the core group for a new coalition-based project was shaped by past and current network linkages within the community. Several sites used existing coalitions for the required CHCC coalition without much change in membership during the formation stage. For example, one used the board of a family resource center and others used existing broad-based health or community improvement coalitions as the grant-required coalition. Other sites built on existing collaborative structures to form the core of a new coalition, but expanded membership for the CHCC initiative through creation of a youth governance group or a committee devoted to the CHCC initiative. The second and related theme is that the composition of the core group influenced who else joined the coalition, either through affiliation with certain groups within the community or through connections the core group had with the larger community. Members from a core group often used their existing relationships, both personal and organizational, to recruit additional members. Coalition members from one of the rural regions spoke of personal connections to a dynamic coordinator: "*I think [coordinator] is a key component. I can say for myself because [coordinator] is a part of this, I was willing to be a part of this. Because there's a fundamental trust that I have in her as a human being, and her genuineness, and her sincerity, and her vision." *When coalitions chose to expand their membership to engage more youth or grass-roots residents, they often pulled in people who already had relationships with the organizations represented on the coalition. One coalition asked each of its members to bring someone new to the next meeting, for example.

**Table 4:** Themes Related to Contextual Influences on Coalition Membership

Coalition Factor and Related Themes	History of Collaboration	Community Politics and History	Community Norms and Values	Community Demographics and Economic Conditions	Geography
**Coalition Membership**					
The core group for a new coalition-based project is shaped by past and current network linkages in the community.	**X**				
Composition of the core group can be influential in shaping coalition membership through members' connections and reputation in the community.	**X**				
Divisions based on community politics and history can limit who joins community coalitions.		**X**			
Tragic events can motivate participation in coalitions and collaborative efforts.		**X**			
Shared values motivate organizations and individuals to join a coalition.			**X**		
Values of independence, rugged individualism and privacy can limit community members' willingness to join a coalition.			**X**		
Economic conditions that necessitate multiple jobs for certain population groups within the community limit participation from those groups.				**X**	
Coalitions serving diverse communities with multiple cultures, languages and SES levels can struggle to achieve broad representation.				**X**	
Recruitment from diverse community sectors may not be possible in rural communities in which certain sectors do not exist.					**X**
Geographic areas with limited opportunities for youth may experience fewer barriers to engaging youth in governance groups.					**X**
Geographic barriers can limit coalition membership.					**X**

Community politics and history can both motivate and limit coalition membership from certain factions of the community. One community, in particular, was strongly divided by an incident at a local school. Because the coordinator and core group were identified with one side of the division, coalition membership during the formation stage was limited to that part of the community. When the impetus for coalition formation came from a certain faction in communities divided in one way or another, coalition membership tended to be limited to groups who shared the goals of the coalition. A second theme in this contextual domain was that past events such as natural disasters, youth violence and economic decline were viewed as motivating people to join community-building efforts. One coalition member explained why people were engaged with their local collaborative initiative: "*Because of the fire, too, because of economics. I think it was a way that we all could connect. We all were affected by the fire or the economic suppression that we have in our community*."

Community norms and values also influenced coalition membership. Widely shared values in a community can help to engage individuals and organizations in a coalition that shares those values. The shared values of helping children, collaborating and creating a sense of community were all mentioned as helping to engage community members in the CHCC coalitions. One community leader explained:

*.. there is a recognition in this community that it just isn't the place it used to be....People knew each other, they liked each other, they enjoyed the community, and there was a real sense of being part of the neighborhood. Then it went through this transition phase ...So that sense of community kind of went down the tubes, and it really wasn't there any more....Folks wanted it to be different, they wanted it to be the old way, they wanted to have a sense of community, and a sense of neighborhood. [...] this seemed to be a way of focusing the efforts that folks had, on something tangible that they could work on*.

Not all values facilitated community engagement, however. Strong values of independence, individualism, privacy and resistance to change can limit participation from certain segments of the community. This was most evident in the rural areas where some people chose to live for the express purpose of privacy. Coalition members identified groups who were resistant to change or "old timers" as especially difficult to engage in coalition efforts.

Coalition membership was also affected by community demographics and economic conditions. Economic conditions that necessitated working long hours or multiple jobs for some community members inhibited participation from those groups. This was most often raised in reference to greater Latino involvement. Young families were also viewed as difficult to engage due to competing demands for time. While sites were fairly successful in engaging youth, participants commented on how youth posed special challenges for coalition membership due to lack of transportation to meetings and the continual need for recruitment as youth graduated from high school and left the community for college and/or jobs. One coordinator commented, "*As of last year I had seven Governance Council members graduate and go on to college so I think I was left with like three or four members who were going to be seniors this year so last August I had to completely recruit new members*."

Participants described that engaging people with lower education levels was difficult due to discomfort with joining a coalition for the first time and a lack of familiarity with how business is typically conducted. Cultural and language barriers in very diverse communities also created barriers that inhibited membership from certain groups, again, often Latinos who had recently immigrated. As one coordinator explained, "*I think one of our biggest holes is we don't have much of a Latino presence on the Board, and that is partly because of the lack of Latinos who have that experience, or feel comfortable joining a Board*."

Geography also played a significant role in determining who joined a coalition. This was most noticeable in rural areas where having a full range of community sectors represented was not always possible simply because they did not exist (e.g., media, housing, environment). As a community leader from one of the rural sites explained, "*we just don't have that many organizations, it's more bringing different components of the community together."*

Distance in rural areas was also mentioned as affecting membership by creating difficulties for those who lived far from the meeting locations or who had unreliable transportation to be actively involved. Although distance was a notable barrier for youth involvement (not just in rural areas), representatives from rural sites spoke of how it was relatively easy to involve youth in governance groups because there were so few other opportunities. A youth leader explained why it was easy to involve youth, *"free pizza and their friends were there. It's a rural area, so there isn't that much to do*."

In all types of communities, rural and urban, geographic divisions limited participation on a coalition in a variety of ways. Distinct small towns in rural regions, freeways dividing communities, gated neighborhoods within communities, and county lines were all mentioned as geographic barriers that limited participation in coalitions unless a concerted effort was made to cross these lines. One coordinator explained, "*we have seven distinct neighborhoods, so we have one [member] representing each of those seven distinct neighborhoods, and there's ethnic diversity, there's economic diversity*."

### Coalition Processes and Structures

Coalition processes include communication, decision-making, conflict resolution, or more generally, how business is conducted within a coalition. History of collaboration and community demographics and economic conditions were the two contextual domains with the largest influence on coalition processes (Table 5). When coalitions are comprised of organizations that have collaborated in the past, current coalition processes are shaped by the way work was completed in earlier efforts. For example, one site had employed an outside facilitator to run their meetings in a past project and continued this practice in the new project. A second theme related to coalition processes was that coalitions in communities with limited economic resources sometimes had difficulty in carrying out simple tasks such as making a photocopy or reliably receiving and sending e-mail messages.

**Table 5:** Themes Related to Contextual Influences on Coalition Processes and Structures

Coalition Factor and Related Themes	History of Collaboration	Community Politics and History	Community Norms and Values	Community Demographics and Economy Conditions	Geography
**Coalition Processes**					
When coalitions are comprised largely of organizations that have worked together in the past, coalition processes can be shaped by the way prior work was done.	**X**				
Coalitions in resource-poor communities can experience difficulties carrying out simple operations.				**X**	
**Coalition Structure**					
When coalitions evolve from existing collaborative groups, they can inherit those structures, both formal and informal.	**X**				

Structure refers to how the coalition is organized, including work groups, leadership positions, level of formalization, bylaws and written operating procedures. Of the contextual domains examined, history of collaboration had the most noticeable impact on how coalitions were structured (Table 5). History of collaboration affected coalition structure in that coalitions often inherited existing structures, or lack thereof, when an existing group took on a new initiative. For example, in one community, the CHCC governance group was part of a broader, well-established community coalition that included a very large number of partners. This coalition's structure included several elements, such as well-defined task forces, to accommodate multiple partners and projects, including CHCC. One member stated, "*I sort of look at the Coordinating Council as this hub. It's in the center and...all the sectors around it are supposed to move to make everybody move together. The Healthy Cities is just one little piece of it, but it really engaged, I think, the whole wheel to move forward..."*

## Discussion

Community coalitions are formed within and deeply embedded in communities. Viewing communities as complex and dynamic systems, it is no surprise that coalitions are shaped by external as well as internal forces. Despite widespread acknowledgement of the importance of context, relatively few studies have attempted to document how context impacts coalitions. The current study is one of the first to systematically examine how community context influences coalition development in the formation stage.

Of the five contextual domains examined, history of collaboration influenced the broadest range of coalition factors: lead agency selection, staffing and leadership, coalition membership, and coalition processes and structure. These findings emphasize the importance of personal and organizational networks in coalition formation. Selection of the lead agency and core group, which depend in part on who has collaborated on similar projects in the past, has repercussions through the whole formation process, from staffing decisions to membership composition to how a coalition is structured. This suggests that unless a concerted effort is made to form new relationships or establish new ways of working together, future collaborative efforts will likely exhibit many characteristics of past efforts. This finding is supported by others. For example, in an analysis of the challenges faced by coalitions in a large substance abuse initiative, Kadushin et al. state, "the organizational residue of previous coalitions has a strong effect on the way contemporary coalitions play out" [36]. The strong influence of prior collaborations, and the fact that so many CHCC coalitions formed out of, or were situated within, existing collaborative relationships also raises the question of how to classify coalitions by stage. To what extent does refocusing, reconstituting membership, or formalizing loose collaborative relationships define a coalition as in the formation stage?

The geography of a community influenced the formation stage largely through coalition membership and staffing. Barriers such as distance in rural areas or interstates and gated communities in urban areas made it more difficult to obtain broad geographic representation on coalitions. In rural areas, the small population limited the number of agency professionals and residents who could be hired with the needed skill sets to staff a coalition-based effort, thus necessitating a heavier investment of volunteer time from coordinators and coalition members. Other studies have examined a few aspects of geography with respect to coalition formation and operations. Wandersman and colleagues described how geographic distance and a historical rivalry between two small towns created challenges for the establishment of a regional coalition with implications for communication, meeting schedules and coalition structure [15]. Jasuja and colleagues found that size of service region was negatively associated with "community organizational process" which was conceptualized as a series of steps from identifying a target population to evaluation of a specific program [37].

The demographic and economic makeup of a community shaped coalition formation through staffing, coalition membership and infrastructure for coalition operations. Although coordinators were typically connected to the lead agency or core group, project-specific staff were hired with the skills to work in a culturally competent way with specific demographic groups prioritized in the action plan, such as Latinos or adolescents. Economic conditions limited participation from groups who were economically disadvantaged and working two jobs. Establishing a diverse coalition membership, beyond diversity in sector representation, is widely acknowledged as difficult [14,38]. Similar to our finding that a lack of familiarity with how coalitions operate can be a barrier to coalition membership for some demographic groups, McLeroy and colleagues discuss the hierarchical and formal nature of many coalitions as a possible obstacle [14]. Other studies have noted how historical racial tensions and distrust affected coalition formation [15,36]. Although mentioned in a couple of the sites, it did not emerge as a consistent cross-site theme in our examination of the formation stage of coalitions.

The remaining contextual domains, community politics and history, and community norms and values, influenced coalition formation largely by facilitating or inhibiting membership from certain segments of the community. Both domains facilitated membership, for example, by serving as a catalyst for change and contributing to a shared vision or mission for a better future. Both also inhibited community participation. Politics or historical events served to divide communities, and when these divisions were deep, only one side joined the coalition, at least in the early stages of the coalitions. Other studies of context report similar findings [15,36]. Certain values also limited participation from the groups that held them, such as individualism and privacy.

This study has several limitations which should be considered when interpreting the findings reported here. First, only eight communities were included in this study. Although they were selected to represent a range of community types, our findings cannot be generalized to other communities without additional replication. Instead, they should be viewed as an initial step in building an evidence-base to help us understand the role of context in community collaborative efforts. Second, our data sources were limited to coalition members, leaders and staff. Thus, views of the community were presented through their perspectives and may be biased given their shared commitment to a broad view of health, collaboration and community improvement. Third, this was a secondary analysis of data collected for a different purpose. Participants were not asked to reflect on how community context influenced the various constructs in CCAT. The interviews and focus group guides did, however, ask about the factors that facilitated and inhibited the community development process and responses often touched on contextual features of their environments. The likely result of this approach is that context influences coalition formation in many more ways than are presented here. Also, given the sheer volume of qualitative data available for each site, only cross-site themes identified in at least two communities and agreed upon by two members of the research team are reported here. Many more contextual findings applied to only one site.

Keeping these caveats in mind, it is interesting to note the possible implications of this study for CCAT. First, our findings confirm that the five domains we examined have an impact on community coalitions. We also observed that coalition membership may be the most sensitive to community context as we documented evidence of each contextual domain influencing membership. In contrast, lead agency and coalition structure were only influenced by history of collaboration. While it is difficult and perhaps premature to develop specific hypotheses for how context influences coalitions, our study points out possible patterns that should be confirmed or expanded upon in future coalition research.

## Conclusions

In a recent review of research in the field of community psychology, Trickett advocates the use of context as a central organizing concept [39]. This approach has particular salience for coalition research. Coalitions are notoriously difficult to study, in part because they are deeply embedded in unique communities. Case studies that can account for context provide a reasonable methodology for beginning to understand the range of ways the environment can influence coalition functioning and outcomes. As case study findings accumulate, the use of a common theoretical framework, or at least commonly accepted coalition constructs, would facilitate building an evidence-base in this important area of community practice and research.

## Competing interests

The authors declare that they have no competing interests.

## Authors' contributions

MK conceptualized the study, participated in data analysis, and drafted the manuscript. JR and SH helped conceptualize the study, participated in data analysis and identification of themes, and edited the entire manuscript. All authors read and approved the final manuscript.

## Pre-publication history

The pre-publication history for this paper can be accessed here:

http://www.biomedcentral.com/1471-2458/10/90/prepub

## References

[B1] Butterfoss F Goodman R Wandersman A Community coalitions for prevention and health promotion Health Educ Res 1993 8 3 315 330 10.1093/her/8.3.315 10146473

[B2] Wolff T Community coalition building--Contemporary practice and research Am J Community Psychol 2001 29 2 165 172 10.1023/A:1010314326787 11446274

[B3] Roussos S Fawcett S A review of collaborative partnerships as a strategy for improving community health Annu Rev Public Health 2000 21 369 402 10.1146/annurev.publhealth.21.1.369 10884958

[B4] Kreuter M Lezin N Young L Evaluating community-based collaborative mechanisms: Implications for practitioners Health Promot Pract 2000 1 1 49 63 10.1177/152483990000100109

[B5] Foster-Fishman P Berkowitz S Lounsbury D Jacobson S Allen N Building collaborative capacity in community coalitions: A review and integrative framework Am J Community Psychol 2001 29 2 241 261 10.1023/A:1010378613583 11446279

[B6] Zakocs RC Edwards EM What explains community coalition effectiveness? A review of the literature Am J Prev Med 2006 30 4 351 361 10.1016/j.amepre.2005.12.004 16530624

[B7] Clark N Doctor L Friedman A Lachance L Houle C Geng X Grisso J Community coalitions to control chronic disease: Allies against asthma as a model and case study Health Promot Pract 2006 7 2 13S 22S 10.1177/1524839906287982 16636152

[B8] Lasker R Weiss E Miller R Partnership synergy: a practical framework for studying and strengthening the collaborative advantage Milbank Q 2001 79 2 179 205 10.1111/1468-0009.00203 11439464 PMC2751192

[B9] Cramer M Atwood J Stoner J A conceptual model for understanding effective coalitions involved in health promotion programming Public Health Nurs 2006 23 1 67 73 10.1111/j.0737-1209.2006.230110.x 16460423

[B10] Butterfoss FD Kegler M DiClemente R, Crosby R, Kegler M The Community Coalition Action Theory Emerging Theories in Health Promotion Practice and Research 2009 2 San Francisco CA: Jossey-Bass 237 276

[B11] Wolff T A practitioner's guide to successful coalitions Am J Community Psychol 2001 29 2 173 191 10.1023/A:1010366310857 11446275

[B12] Lackey J Welnetz T Balistrieri M Ensuring a prevention presence when coalitions falter: A case study of a community coalitions demonstration project Eval Program Plann 2000 23 355 363 10.1016/S0149-7189(00)00024-0

[B13] Wells R Ford E Holt M McClure J Ward A Tracing the evolution of pluralism in community-based coalitions Health Care Manage Rev 2004 29 4 329 343 15600111 10.1097/00004010-200410000-00009

[B14] McLeroy K Kegler M Steckler A Burdine J Wisotzky M Community coalitions for health promotion: Summary and further reflections Health Educ Res 1994 9 1 1 11 10.1093/her/9.1.1 10146731

[B15] Wandersman A Valois R Ochs L de la Cruz D Adkins E Goodman R Toward a social ecology of community coalitions Am J Health Promot 1996 10 4 299 307 10172711 10.4278/0890-1171-10.4.299

[B16] Granner M Sharpe P Evaluating community coalition characteristics and functioning: a summary of measurement tools Health Educ Behav 2004 19 5 514 32 10.1093/her/cyg056 15150134

[B17] Florin P Mitchell R Stevenson J Klein I Predicting intermediate outcomes for prevention coalitions: A developmental perspective Eval Program Plann 2000 23 341 346 10.1016/S0149-7189(00)00022-7

[B18] Downey L Ireson C Slavova S McKee G Defining elements of success: A critical pathway of coalition development Health Promot Pract 2008 9 2 130 139 10.1177/1524839907311573 18340088

[B19] Chaskin R Chipenda-Dansokho S Toler A Moving Beyond the Neighborhood and Family Initiative: The Final Phase and Lessons Learned 2000 Chicago, IL: Chapin Hall Center for Children at the University of Chicago

[B20] Butterfoss FD LaChance LL Orians CE Building Allies coalitions: Why formation matters Health Promot Pract 2006 7 23 33S 10.1177/1524839906287062 16636153

[B21] Butterfoss FD Gilmore LA Krieger JW Lachance L Lara M Meurer J Orians C Peterson J Rose S Rosenthal M From formation to action: How Allies Against Asthma coalitions are getting the job done Health Promot Pract 2006 7 34 43S 10.1177/1524839906287063 16636154

[B22] Nezlek J Galano J Developing and maintaining state-wide adolescent pregnancy prevention coalitions: A preliminary investigation Health Educ Res 1993 8 3 433 447 10.1093/her/8.3.433 10146479

[B23] Chilenski S Greenberg M Feinberg M Community readiness as a multidimensional construct J Community Psychol 2007 35 3 347 365 10.1002/jcop.20152 18714368 PMC2517859

[B24] Findholt N Application of the community readiness model for childhood obesity prevention Public Health Nurs 2007 24 6 565 570 10.1111/j.1525-1446.2007.00669.x 17973734

[B25] Ogilvie K Morre R Ogilvie D Johnson K Collins D Shamblen S Changing community readiness to prevent the abuse of inhalants and other harmful products in Alaska J Community Health 2008 33 4 248 58 10.1007/s10900-008-9087-7 18392927 PMC2444046

[B26] McCoy H Malow R Edwards R Thurland A Rosenberg R A strategy for improving community effectiveness of HIV/AIDS intervention design: the Community Readiness Model in the Caribbean Subst Use Misuse 2007 42 10 1579 92 10.1080/10826080701212535 17918028

[B27] Edwards R Jumper-Thurman P Plested B Oetting E Swanson L Community readiness: research to practice J Community Psychol 2000 28 3 291 307 10.1002/(SICI)1520-6629(200005)28:3<291::AID-JCOP5>3.0.CO;2-9

[B28] Feinberg M Greenberg M Osgood D Readiness, functioning and perceived effectiveness in community prevention coalitions: A study of Communities That Care Am J Community Psychol 2004 33 3/4 163 176 10.1023/B:AJCP.0000027003.75394.2b 15212176

[B29] Reininger B Dinh-Zarr T Sinicrope P Martin D Dimensions of participation and leadership: Implications for community-based health promotion for youth Fam Community Health 1999 22 2 72 82

[B30] Kegler M Steckler A Malek S McLeroy K A multiple case study of implementation in 10 local Project ASSIST coalitions in North Carolina Health Educ Res 1998 13 2 225 238 10.1093/her/13.2.225 10181021

[B31] Kegler M Twiss J Look V Assessing community change at multiple levels: The genesis of an evaluation framework for the California Healthy Cities and Communities Project Health Educ Behav 2000 27 6 760 779 10.1177/109019810002700610 11104374

[B32] Kegler M Norton B Aronson R Achieving organizational change: Findings from case studies of 20 California Healthy Cities and Communities coalitions Health Promot Int 2008 23 2 109 18 10.1093/heapro/dan009 18359785

[B33] Patton M Qualitative Research and Evaluation Methods 2002 Third Thousand Oaks, CA: Sage Publications

[B34] Yin R Applications of Case Study Research 1993 Thousand Oaks, CA: Sage Publications

[B35] Yin R Case Study Research: Design and Methods 2003 3 Thousand Oaks, CA: Sage Publications

[B36] Kadushin C Lindholm M Ryan D Brodsky A Saxe L Why it is so difficult to form effective community coalitions City and Community 2005 4 3 255 275 10.1111/j.1540-6040.2005.00116.x

[B37] Jasuja G Chou C Bernstein K Wang E McClure M Pentz M Using structural characteristics of community coalitions to predict progress in adopting evidence-based prevention programs Eval Program Plann 2005 28 173 184 10.1016/j.evalprogplan.2005.01.002

[B38] Kaye G Grassroots involvement Am J Community Psychol 2001 29 2 269 275 10.1023/A:1010382714491 11446281

[B39] Trickett E Community psychology: Individuals and interventions in community context Annu Rev Psychol 2009 60 395 419 10.1146/annurev.psych.60.110707.163517 19035828

